# 

**Published:** 1878-05

**Authors:** 


### CONTENTS.

### COMMUNICATIONS.

Address.—*By Geo. L. Field, D. D. S.* ........ 177

Address.—*By Dr. Jas. Taylor* ........ 184

Mechanical Dentistry.—*By J. B. Tullis, D. D. S.* ........ 190

Address.—*By H. E. Beach, D. D. S.* ........ 192

### PROCEEDINGS OF SOCIETIES.

Discussions of the Mississippi Valley Dental Association ........ 195
The Thirty-Fourth Annual Meeting of the Mississippi Valley Association of Dental Surgeons ........ 205

### MISCELLANEOUS.

Correspondence ........ 208
Another American Dentist for Europe ........ 211
Nerve Stretching for the Relief of Neuralgia ........ 211

### EDITORIAL.

"Do we use Diamond Disks?" ........ 213
Kentucky State Dental Society ........ 213
Iowa State Dental Society ........ 215
A Law Regulating the Practice of Dentistry in Kentucky ........ 217
Portland Cement a Material for Filling.—*By Dr. Witte,* Hanover ........ 219
From the Vierteljahrsschrift ........ 220

